# Moving developmental social neuroscience toward a second-person approach

**DOI:** 10.1371/journal.pbio.3000055

**Published:** 2018-12-13

**Authors:** Stefanie Hoehl, Gabriela Markova

**Affiliations:** 1 Faculty of Psychology, University of Vienna, Vienna, Austria; 2 Max Planck Institute for Human Cognitive and Brain Sciences, Leipzig, Germany

## Abstract

Infants’ cognitive development and learning rely profoundly on their interactions with other people. In the first year, infants become increasingly sensitive to others’ gaze and use it to focus their own attention on relevant visual input. However, infants are not passive observers in early social interactions, and these exchanges are characterized by high levels of contingency and reciprocity. Wass and colleagues offer first insights into the neurobehavioral dynamics of caregiver–infant interactions, demonstrating that caregivers’ scalp-recorded theta band activity responds to their infant’s changes in attention, and parental brain activation is associated with infants’ sustenance of attention. This research opens up entirely new ways of exploring caregiver–infant interactions and to understand early social attention as a reciprocal and dynamic process.

## Abstract 2

Developmental social neuroscience has considered infants mostly as passive observers, despite their active engagement with other people in their daily lives. This Primer explores an important new step towards a better understanding of the neurobehavioral dynamics of naturalistic caregiver-infant interactions.

## Moving developmental social neuroscience toward a second-person approach

Developmental research on social attention in infancy has provided insights on the impact of early gaze following on infant learning and, somewhat later in development, their active direction of other peoples’ attention in order to share experiences and information with them [1]. Yet this research has largely utilized experimental setups in which only infants’ behavior and/or neural responses were recorded. A significant knowledge gap exists regarding the neurobehavioral dynamics of caregiver–infant exchanges that, in real life, are reciprocal and entail a complex interplay of caregivers’ and infants’ fluctuating attention and behavior. These dynamics are difficult to capture in laboratory studies, and recording infant brain activity poses a serious methodological challenge, even in conventional experimental paradigms. New approaches are now being developed to tackle this “dark matter” of developmental social neuroscience.

## The “dark matter” of social neuroscience

The standard neuroscientific approach to examine social attention and understanding of others is to study an individual’s ability to process social information detached from any social context. That is, traditional social neuroscience predominantly examines neural correlates of social perception “from an observer’s point of view.” This approach leaves us with open questions about the neural underpinning of truly social, reciprocal engagements with other individuals—what Schilbach and colleagues dubbed the “dark matter” of social neuroscience [2]. The importance of interpersonal interactions for understanding others is highlighted by enactive and embodied approaches to studying social processes [3]. This second-person approach is based on the idea that being and engaging with others is fundamental to comprehending them. Only within truly social exchanges can we fully grasp other people’s emotions, intentions, desires, and thoughts because within second-person engagements, we are directly addressed and responded to by another mind [2].

Interestingly, the importance of close, emotional engagements with other people has been extensively studied in behavioral developmental psychology (see “Synchrony and reciprocity in caregiver–child interactions” below), and the importance of direct engagement for infants’ social learning has been recognized [4]. Yet developmental social neuroscience research almost exclusively relies on paradigms in which infants passively observe artificial stimuli on a computer screen. This discrepancy between infants’ day-to-day experiences and experimental paradigms is evident in research on early social attention.

## Development of social attention in infancy

The least ambiguous signal of whether one is “being-with” others may be through eye gaze. In fact, gaze not only determines what is socially relevant to the infant in the environment [5,6] but may also allow infants to experience themselves as the object of others’ attention [7]. Infants’ attention in social interactions has been the topic of extensive research and debate ever since Scaife and Bruner [8] demonstrated young infants’ ability to align their gaze with another person’s line of regard. The vast majority of research on the development of social attention has explored infants’ looking behavior in response to another person’s gaze direction. Newborns are already sensitive to others’ gaze [9], and from around three months of age, they overtly follow another person’s gaze toward items that are close by [10]. Over the course of the first year, infants’ gaze following becomes more precise [11] and sophisticated, with an increasing ability to follow gaze toward targets located further away and hidden from immediate sight [12,13]. Between 10 and 12 months of age, infants gain an understanding that the eyes are critical for seeing, and they consequently stop following the head direction of a person with occluded or closed eyes [14]. Around the same age, infants start using declarative pointing gestures to direct others’ attention toward interesting sights in the environment [15], indicating that, by this age, they recognize others as intentional agents with points of views that can be shared [16]. In developmental psychology, these aspects of social attention, gaze following on the one hand and directing others’ attention on the other hand, have been conceptualized as responding to and initiating joint attention, respectively [17]. The summarized findings demonstrate that behavioral research was highly successful in delineating the developmental trajectory of social attention in infancy. Yet infants’ behavior (i.e., gaze following and pointing) offers limited insight into the online cognitive processes that determine how and where infants focus their attention.

Addressing the underlying mechanisms of early social attention, neuroscientific research on gaze processing has largely relied on presenting infants with pictures or videos featuring people or virtual characters gazing toward target objects near the face [18–20] or correlating baseline electroencephalography (EEG) coherence measures with infants’ behavioral skills in live interactions [21]. Findings suggest that, similar to adults, separable brain regions are involved in responding to and initiating joint attention [17], some of which show sensitivity to gaze cues as early as at four–five months of age [19,22]. In addition, by four months, infants’ attention is not only effectively guided by others’ gaze [23] but gaze cues also enable more effective encoding of cued objects compared to distractor objects [24–26]. These experiments show that infants’ visual information processing is heavily influenced by adults’ social attention cues, but they leave open how attention is regulated in dynamic exchanges between infants and their adult caregivers. Though some recent studies measured infants’ electrophysiological brain activities during live social interactions [6,27–29], exchanges in these studies were highly controlled, thus lacking natural timing, and (with one exception [29]) did not include neurobehavioral assessments of the adult interaction partner. This is despite the fact that extensive evidence points to the importance of synchrony and reciprocity in early social exchanges.

## Synchrony and reciprocity in caregiver–child interactions

From early in life, infants recognize and create structures in their engagements with others. Early social interactions between infants and their caregivers are mutually regulated systems that are characterized by a face-to-face context, close physical contact, and a turn-taking structure, in which social engagement and disengagement alternate [30,31]. Early social interactions can thus be considered a mutual, bidirectional process [32], in which both partners modulate the timing, the form, and the intensity of the interaction and their own emotional expression to achieve complementary interactive exchanges [33] and to share meaning, particularly emotions, with the other person [34,35]. Consequently, infants are also greatly sensitive to disturbances to these mutually regulated, emotional interactions, as can be observed in their interactions with withdrawn and/or disengaged caregivers [36,37]. This research on the consequences of maternal unavailability suggests that infants have particular expectations of others’ behaviors when engaging in social interactions with them and react with negative affect when their expectations are being violated.

Behavioral evidence suggests that in these dynamic engagements with others, infants coordinate their actions with those of their caregivers. This so-called interpersonal synchrony is a defining and probably involuntary feature of early social exchanges (see also [38]). In fact, even neonates seem to be able to synchronize their behaviors with those of adults [39], and most recent evidence suggests that interpersonal synchrony has a biological basis [40]. Most prominently, research has shown that the neuropeptide oxytocin is a physiological mechanism underlying synchronous exchanges between infants and their caregivers [41]. Furthermore, recent research with adults using simultaneous recordings of brain activities from several persons (i.e., hyperscanning methods) suggests that interactive synchrony also manifests as interpersonal coupling of brain activities [42–44]. However, the neural mechanisms of early social engagements largely remain “in the dark.”

## Parental neural responsivity to infants’ visual attention

The study by Wass and colleagues [45] constitutes an important step in the direction of more ecologically valid paradigms for studying early social attention and toward elucidating the brain mechanisms underlying dynamic caregiver–infant exchanges. Wass and colleagues recorded dual-EEG from 12-month-old infants and their primary caregivers while both were playing with an object either together (joint play) or alone (solo play). At the same time, looking behavior from infant and caregiver were monitored and video-coded offline. Importantly, this approach not only allows for tracking infants’ and adults’ brain activities during a naturalistic interaction but it also provides first insights into how each partner influences the other during a reciprocal and dynamic exchange. Their results, reported in this issue of *PLOS Biology*, show that infant gaze is an important communication signal for parents, enabling coordination at the behavioral and the neural level. In particular, the caregiver’s scalp-recorded theta power was found to increase after infants’ look toward the object during joint play. Furthermore, infants looked longer at the objects (indicating longer sustained attention) the more their caregiver’s theta power increased. Thus, increased parental neural responsiveness to their infant’s gaze behavior predicted the infant’s duration of looking toward a jointly attended object. These findings offer first clues as to how caregivers’ engagement in social interactions may affect infants’ attention control and, thus, pave the way to a deeper understanding of infant social attention in naturalistic exchanges.

Interestingly, Wass and colleagues also found that infants have more endogenous control over their attentional processes when playing alone than with their caregiver. Taken together, this set of findings opens up further interesting questions that remain unanswered by this study. For example, the relationship between infants’ abilities and parental control remains unclear. Specifically, are there individual differences in infants’ endogenous attentional control that may be attributed to caregiving quality or particular characteristics of the dyad? In this context, examining interindividual variations in caregiver neural responsiveness may reveal important factors that codetermine the relationship between infants’ own attention control and parental coregulatory abilities. It would also be interesting to ask whether infants come to expect and anticipate parental attentional scaffolding and what happens when parental responsiveness is lowered, e.g., due to a psychopathological condition, or modified by ineffective social capabilities of the infant. In fact, Leclére and colleagues [46] suggest that effective coordination of behavior among parent–child dyads is associated with a healthy parent and typically developing child. It is conceivable that similar factors affect neural coregulation of attention, as examined by Wass and colleagues. Last but not least, the development of coordination and regulation abilities as well as their developmental consequences remain subject to future research in this field and could not be addressed in this cross-sectional investigation.

Notably, Wass and colleagues collected dual-EEG data during naturalistic exchanges between infants and their parents. As infants cannot be instructed to minimize movements and as a certain degree of movement is mandatory to allow for dynamic social exchanges, this approach is highly challenging. The authors dealt with these problems by limiting their analyses to the recording channels and frequency ranges that are the least affected by motor artifacts, though not completely unaffected. Nevertheless, artifacts pose a serious challenge to EEG live interaction studies, requiring careful consideration and limiting the scope of what can be meaningfully addressed in developmental social neuroscience by using EEG measures.

## Future directions

**Fig 1:**
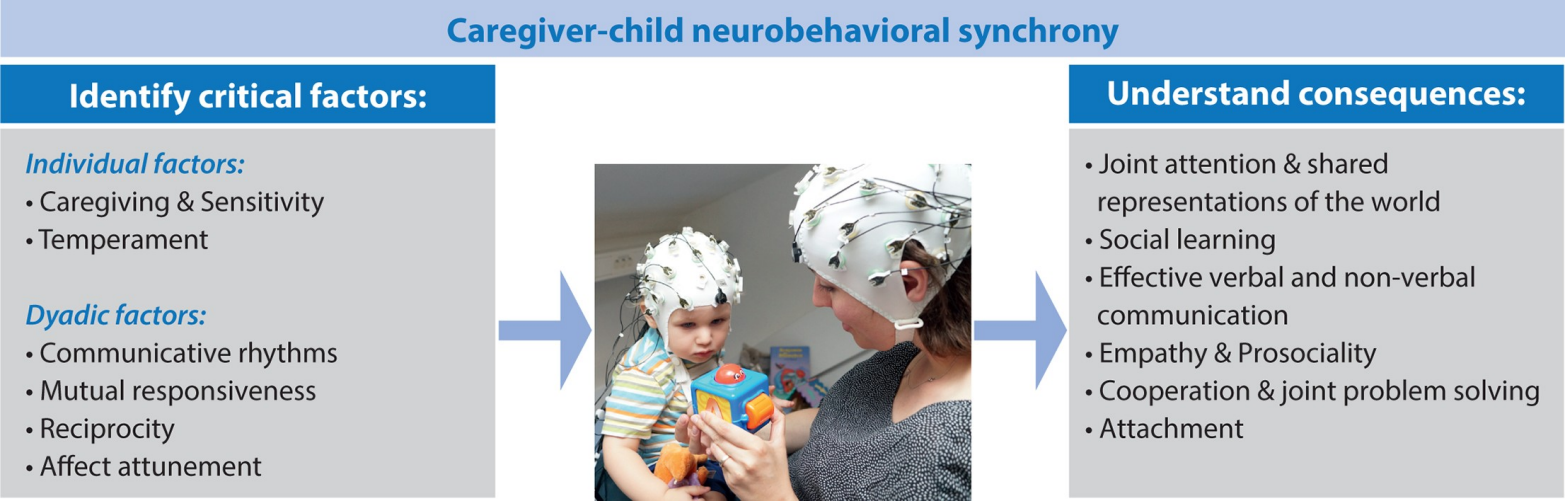
Future directions in developmental social neuroscience regarding the causes and consequences of neurobehavioral synchrony in caregiver–child interactions.

The study by Wass and colleagues [45] shows that combining naturalistic live interactions between infants and their caregivers with measures of brain activity is a challenging yet feasible approach with the potential of moving developmental social neuroscience toward a second-person approach. Importantly, the combination of behavioral with neuroscientific measures seems to be most informative, elucidating the interplay between infant and caregivers’ behaviors. There are numerous future directions for this line of research (see Fig 1), of which we highlight three that seem especially promising.

In line with previous research demonstrating increased infant attention toward objects during joint attention [27], Wass and colleagues report that infants looked longer at the objects during joint play versus solo play and that the durations of infants’ looks were associated with their caregiver’s neural responses. This likely has important implications for infants’ social learning. Future research will look into the dynamics of social learning interactions in which infants acquire knowledge about, e.g., the affective value and function of objects. As behavioral contingency plays a vital role for language acquisition [4,47], dual-brain recording during verbal interactions (not necessarily using EEG due to speech-related artifacts) may further provide invaluable insights into how caregivers’ behavioral and neural responses to infant speech shape early vocalizations.

In addition, caregiver–infant behavioral and physiological synchrony has been proposed as fundamental to early attachment formation [40]. While neuroimaging research has helped to delineate the neuroanatomical networks involved in the perception and regulation of social information depending on an individual’s attachment style in adults [48], we have limited knowledge of the neural mechanisms of attachment formation in early childhood. Hyperscanning may provide important insights into how the neurobehavioral dynamics of infant–caregiver exchanges relate to later attachment security in the child. In this context, it will also be illuminating to address how neurobehavioral synchrony in parent–child interactions is obtained and to evaluate its functions through modulating interpersonal synchrony, e.g., through communicative or external perceptual rhythms or by perturbing infant–caregiver interactions.

Finally, it seems important to point out that human social interactions, especially between caregiver and child, rely on a multitude of rhythms, including auditory rhythms such as speech (syllables, phonemes, and prosody) and singing, motor action rhythms, tactile rhythms (i.e., affective or stimulating touch), as well as cardiac pulsation and respiration rhythms, all of which can synchronize in close physical proximity [49]. While we are beginning to understand how different physiological and brain rhythms are interrelated within adult individuals [50], we have yet to grasp the mechanisms and functions of interpersonal rhythms on the behavioral, physiological, and neural level and how they relate to each other in early caregiver–child interactions.

## Abbreviation

EEG: electroencephalography
